# Modulation of haemodynamics, endogeneous antioxidant enzymes, and pathophysiological changes by selective inhibition of angiotensin II type 1 receptors in pressure-overload rats

**DOI:** 10.5830/CVJA-2012-080

**Published:** 2013-04

**Authors:** Ghulam Moinuddin, Mohammed Naseeruddin Inamdar, Kala S Kulkarni, Chanda Kulkarni

**Affiliations:** Department of Pharmacology, Al-Ameen College of Pharmacy, Bangalore, India; Department of Pharmacology, Al-Ameen College of Pharmacy, Bangalore, India; Clinical Pharmacy, School of Pharmacy and Technology Management, Mumbai, India; Department of Pharmacology, St John’s Medical College, Bangalore, India

**Keywords:** abdominal aortic banding, cardiac hypertrophy, hypertension, RAS, AT1 receptor blockers

## Abstract

**Background:**

Constriction of the thoracic or abdominal aorta provides an experimental model of pressure-overload cardiac hypertrophy. Blockade of AT_1_ receptors is beneficial in preventing target-organ damage in hypertension.

**Objective:**

To examine the effect of angiotensin II receptor antagonists on blood pressure, endogenous antioxidant enzyme and histopathological changes in pressure-overload rats.

**Methods:**

Pressure overload was produced by abdominal aortic banding (AAB) using a blunt 22-guage needle in male rats as a model of cardiac hypertrophy. After surgery, the AAB-induced hypertension (AABIH) rats were treated with losartan 40 mg/kg/day, candesartan 10 mg/kg/day, irbesartan 10 mg/kg/day per os for 16 weeks. At 16 weeks of surgery, the rats were observed for general characteristics and mortality, and we determined non-invasive blood pressure (NIBP), endogenous antioxidant enzyme catalase and superoxide dismutase (SOD) activities, and histology of the target organs.

**Results:**

In the AABIH group, significant increase in systolic blood pressure was observed from weeks 3 to 16 compared with the control group, along with reduced serum catalase and SOD activities. The treated groups showed significant reduction in systolic BP and increase in serum SOD and catalase activities. The histological changes induced in the target organs, namely heart, liver, kidneys and thoracic aorta in the AABIH rats were attenuated in the treated rats.

**Conclusion:**

Blockade of the AT_1_ receptor caused an improvement in the myocardial antioxidant reserve and decreased oxidative stress in the hypertensive rats, which was evidenced by the protection observed in the treatment groups.

## Abstract

Angiotensin II (Ang-II), the most active component of the renin–angiotensin system (RAS), is a multifunctional hormone that plays an important role in cardiovascular physiology and pathology.^1^ Ang-II production in proximity to its receptors on the target cells constitutes the local RAS, which regulates cardiovascular functions in both autocrine and paracrine systems. Ang-II, a potent mediator of the RAS, plays a pivotal physiological role in cardiovascular homeostasis. Ang-II is a strong vasoconstrictor of the peripheral vasculature and induces hypertrophy, hyperplasia or both in resistance arteries, vascular smooth muscle cells (VSMCs), endothelial cells and cardiomyocytes.^2^-^5^

Due to these actions, Ang-II is thought to be an important mediator in the development and maintenance of hypertension, atherosclerosis, diabetes, and cardiac and renal failure.^6^ The actions of Ang-II are primarily mediated by two receptors, Ang-II type 1 (AT_1_) and type 2 (AT_2_). The activation of the AT_1_ receptor mediates vasoconstriction, proliferation of vascular smooth muscle cells, and production of extracellular matrix proteins by vascular smooth muscle cells. By contrast, the AT_2_ receptor has been considered to mediate vasodilation, antiproliferation, and pro-apoptosis in the vasculature, presumably mediated by the activation of the nitric oxide (NO) system via bradykinin production.^1^

The elevation of systemic blood pressure (BP) associated with hypertension is a risk factor for cardiovascular disease and renal failure. Often it is the pathophysiological alterations and impairments associated with hypertension that lessen life expectancy. Pharmacological intervention has been relatively successful in normalising the elevation in BP. However, the assumption that reduction in BP will totally reverse hypertension-induced pathophysiological changes remains unclear.^7^-^10^

Cardiac hypertrophy is an increase in the mass of the contractile and ancillary proteins of the heart above that which is normal for the given stage of its maturational growth.^11^ In its initial stages, the hypertrophied ventricle is able to compensate in the face of an increased workload, but in later stages, the diastolic and eventually the systolic properties of the left ventricle become impaired, causing decompensation, which this leads to heart failure. The commonest cause of cardiac hypertrophy is hypertension. Hypertrophy is an independent risk factor for sudden death of unknown origin and also increases the risk of myocardial ischaemia and ventricular arrhythmias.^11^

A role for the RAS in the development of hypertension is well established in both human and animal models, such as the spontaneously hypertensive rat. Interruption of the RAS pathway, either by preventing the formation of Ang-II (i.e. angiotensin-converting enzyme inhibitor) or by blocking its actions at the level of the peptide receptor (AT1 receptor antagonists), has been shown to reduced BP and protect against target-organ injury.^12^-^15^ However, the attenuation or delay of non-haemodynamic pathophysiological impairments with these agents does not reduce the risk in hypertensive patients.^9^-^10^ In addition, chronic administration of traditional therapies is necessary for long-term antihypertensive benefits.

Constriction of the thoracic or abdominal aorta provides an experimental model of what has previously been described as pressure-overload cardiac hypertrophy. The increased blood pressure proximal to the constriction has been postulated to provide a stimulus for the development of cardiac hypertrophy.^16^

This study was designed to examine the effects of AT_1_ receptor antagonists on the non-invasive (indirect) tail-cuff method, using an automated cuff inflator pulse-detection system to estimate the endogenous antioxidant enzyme [serum catalase and superoxide dismutase (SOD)] activity. Histopathological changes in the target organs (heart, liver, kidneys and thoracic aorta) were analysed to compare the histopathological changes induced in untreated abdominal aortic banding-induced hypertension (AABIH) and cardiac hypertrophy in rats.

## Methods

Healthy adult male albino Wistar rats weighing between 150 and 210 g were selected. Animals were maintained under standard laboratory conditions at 28 ± 2°C, relative humidity of 50 ± 15% and normal photo-period (12-h dark and 12-h light). Commercial pellet diet (Amruth Ltd, India) and water were provided *ad libitum*.

The experimental protocol was approved by the Institutional Animal Ethics Committee and by the animal regulatory body of the government (Al-Ameen College of Pharmacy, India. Reg. No. 83/1999/CPCSEA). The test drugs losartan, candesartan and irbesartan were procured from Micro Labs Private Ltd and Biocon Ltd, India, respectively.

Animals were randomly divided into different groups, each with eight male Wistar rats and they were treated as follows: control (normotensive) sham-operated rats; untreated AABIH rats; AABIH rats treated with losartan (40 mg/kg/day p.o.); candesartan (10 mg/kg/day p.o.); and irbesartan (10 mg/kg/day p.o.), respectively. Pressure overload was produced by abdominal aortic banding (AAB), which has primarily been used as a model of cardiac hypertrophy.^17^

Briefly, animals were anesthetised using a combination of ketamine (70 mg/kg, i.p) and xylazine (10 mg/kg, i.p.) and the aorta was exposed through a midline abdominal incision. For the banding model, a blunt 22-gauge needle was placed adjacent to the abdominal aorta between the renal arties just below the renal bifurcations, and a ligature was tightened around the aorta and adjacent needle. The sham procedure for the control rats included injection of the same dose of combination anesthesia, an incision of approximately the same size, and the placement of a loosely tied ligature at the same position on the abdominal aorta.^18^ The muscular layer was sutured, followed by the abdominal skin, and the animals were isolated in a cage for recovery. The dead animals were removed from the cage.

Drug treatment was started on the animals recovering from surgery, with losartan, candesartan and irbesartan administered daily for 16 weeks. The three drugs were formulated freshly using 1% carboxy methyl cellulose (CMC) in distilled water and were administered orally in a dose volume of 2 ml/kg body weight; 1% CMC solution was used as vehicle.

After the surgery the animals were placed in their cages and were observed for general characteristics and mortality. Non-invasive (indirect) blood pressure (NIBP) was determined by the tail-cuff method using an automated cuff inflator pulse-detection system (AD Instruments, NIBP measurement apparatus).

Non-anaesthetised rats were placed in a restraining holder from which the tail protruded. Vasodilatation was achieved by local warming of the tail with an infrared bulb. The cuff and transducer were placed around the tail, and the cuff was inflated until the pulse disappeared. When the cuff was deflated, the point of reappearance of the pulse indicated the value of systolic blood pressure. The reported values are from a minimum of three recordings performed on each animal by the same investigator. The NIBP was measured during weeks 1, 3 and 16. The patency of the hypertension induced by AAB was ascertained during week 3.

## Endogenous anti-oxidant enzyme activity

After the NIBP measurement, the rats were anaesthetised with ether and blood was collected in 2-ml Eppendorff tubes from the retro-orbital plexus, with the help of heparinised capillary tubes, for the estimation of anti-oxidant enzyme activity. The collected blood was centrifuged for 15 min at 7 000 rpm and the supernatant (serum) was used for the estimation of biochemical parameters, namely catalase and SOD activity.

The catalase activity was determined spectrophotometrically according to standard protocol as per the Clariborne method.^19^ Briefly, to 1.95 ml of 10 mM H_2_O_2_ in 60 mM phosphate buffer (pH 7.0), 0.05 ml of the plasma/serum was added and degradation of H2O2 was followed at 240 nm per min. The rate of decomposition of H_2_O_2_ was calculated using the formula *k* = 2.303/Δt × log (A_1_/A_2_) S^-1^, followed by calculation of catalase in terms of U/mg of protein. A unit of catalase is defined as the quantity that decomposes 1.0 μmole of H_2_O_2_ per min at pH 7.0 and 25°C, while this H2O2 concentration falls from 10.3 to 9.2 mM.

SOD activity was determined based on the ability of SOD to inhibit the auto-oxidation of epinephrine to adrenochrome at alkaline pH as per the method of Misra and Fridovich.^20^ Briefly, 25 μl of the supernatant obtained from the centrifuged blood was added to the mixture of 0.1 mM adrenaline in carbonate buffer (pH 10.2) in a total volume of 1 ml, and the formation of adrenochrome was measured at 295 nm. The SOD activity (U/mg of protein) was calculated using a standard plot.

## Histopathological evaluation of target organs

At the end of 16 weeks, after the NIBP measurement, rats from each group were anaesthetised with ether and the target organs (heart, liver, kidneys and thoracic aorta) were collected and placed in the separate containers containing 10% neutral buffered formalin, pH 6.8–7.0 (10 ml 40% formaldehyde, 0.35 g anhydrous sodium dihydrogen phosphate, 0.65 g anhydrous disodium hydrogen phosphate, 90 ml distilled water). The samples were sectioned, stained and processed for histopathological evaluation. The organs were processed, sectioned at 5-μm thickness and stained with standard haematoxylin and eosin. The slides were mounted and evaluated under a microscope by a qualified pathologist.

The histological evaluation was performed to compare the changes induced in untreated and treated AABIH rats with AT_1_ receptor blockers in comparison with the control, sham-operated rat organs (heart, liver, kidneys and thoracic aorta).

## Statistical analysis

The values are expressed as mean ± SEM. Data were analysed by analysis of variance (ANOVA) followed by Tukey’s multiple-comparison test to compare the treatment groups with the control group using a GraphPad Prism.

## Results

The sham-operated control (normotensive) group, AABIH rats, and the groups treated with AT1 receptor antagonists (losartan, candesartan and irbesartan) were monitored periodically. In terms of general appearance and behaviour, nothing unusual was noted in any of the treatment groups. The body weight gain in both the treated and untreated groups was slightly lower than in their respective control groups, but the differences were not significant (*p* > 0.05). Mortality in the AAB animals during or immediately after surgery was about 20%. Another 15% of the animals died within 24 hours of surgery.

In the AABIH group, there was a significant increase in systolic blood pressure from weeks 3 to 16 (*p* < 0.001) compared to the control, sham-operated group. Significant reduction in the systolic blood pressure was observed in the losartan-, candesartan- (*p* < 0.001) and irbesartan-treated (*p* < 0.05) groups, compared with the AABIH group (Table 1).

**Table 1:** Effect Of AT_1_ Receptor Antagonists On Systolic BP Of AABIH And Cardiac Hypertrophy Rats

*Treatment*	*Systolic BP (mmHg) week 1*	*Systolic BP (mmHg) week 16*	*% Increase in systolic BP week 16*
Control	94.14 ± 0.589	105.9 ± 0.7004	12.96 ± 1.21
Hypertensive	114.5 ± 0.816	158.7 ± 2.194	39.37 ± 1.494
	149.3 ± 0.821^#^		
Losartan	114.4 ± 0.9197	120.3 ± 4.113	6.144 ± 3.66***
Candesartan	104.8 ± 1.880	126.0 ± 2.481	14.04 ± 3.98***
Irbesartan	102.8 ± 0.427	126.7 ± 1.298	23.75 ± 0.895*

AABIH = abdominal aortic banding-induced hypertension. Values are expressed in mean ± SEM, *n* = 8. Statistical analysis: one-way analysis of variance (ANOVA) followed by Tukey’s multiple comparison test. *Statistically significant decrease in systolic BP compared with hypertensive group (*p* < 0.05). **Statistically significant decrease in systolic BP compared with hypertensive group (*p* < 0.01). ***Statistically significant decrease in systolic BP compared with hypertensive group (*p* < 0.001). ^#^Systolic blood pressure during week 3. The patency of the hypertension induced by abdominal aortic banding was ascertained during week 3.

In the AABIH group, there was a significant (*p* < 0.001) decrease in catalase activity compared to the control, shamoperated group. In the groups treated with losartan (*p* < 0.001) and irbesartan (*p* < 0.05), there was a significant increase in the level of catalase activity compared with the AABIH group. However, there was no significant increase in catalase activity in the candesartan group (*p* > 0.05) (Table 2).

**Table 2:** Effect Of AT_1_ Receptor Antagonists On Serum Sod And Catalase Levels In The Pressureoverload AABIH And Cardiac Hypertrophy Rats

*Treatment*	*SOD (units /ml)*	*Catalase (units /ml)*
Control	19.61 ± 0.4095	160.0 ± 5.768
Hypertensive	12.92 ± 0.4601	144.7 ± 2.204
Losartan	35.55 ± 1.622***	202.0 ± 3.539***
Candesartan	25.72 ± 1.586***	146.6 ± 1.997
Irbesartan	27.74 ± 0.7738***	176.2 ± 4.043*

AABIH = abdominal aortic banding-induced hypertension. Values are expressed in mean ± SEM, *n* = 8. Statistical analysis: one-way analysis of variance (ANOVA) followed by Tukey’s multiple comparison test. *Statistically significant decrease in systolic BP compared with hypertensive group (*p* < 0.05) **Statistically significant decrease in systolic BP compared with hypertensive group (*p* < 0.01) ***Statistically significant decrease in systolic BP compared with hypertensive group (*p* < 0.001).

In the AABIH group, there was a significant reduction in SOD activity when compared to the control, sham-operated group. A significant increase (*p* < 0.001) in serum SOD activity was observed in the losartan-, candesartan- and irbesartantreated groups, compared to the AABIH group (Table 2).

## Histopathological evaluation

**Fig. 1.**
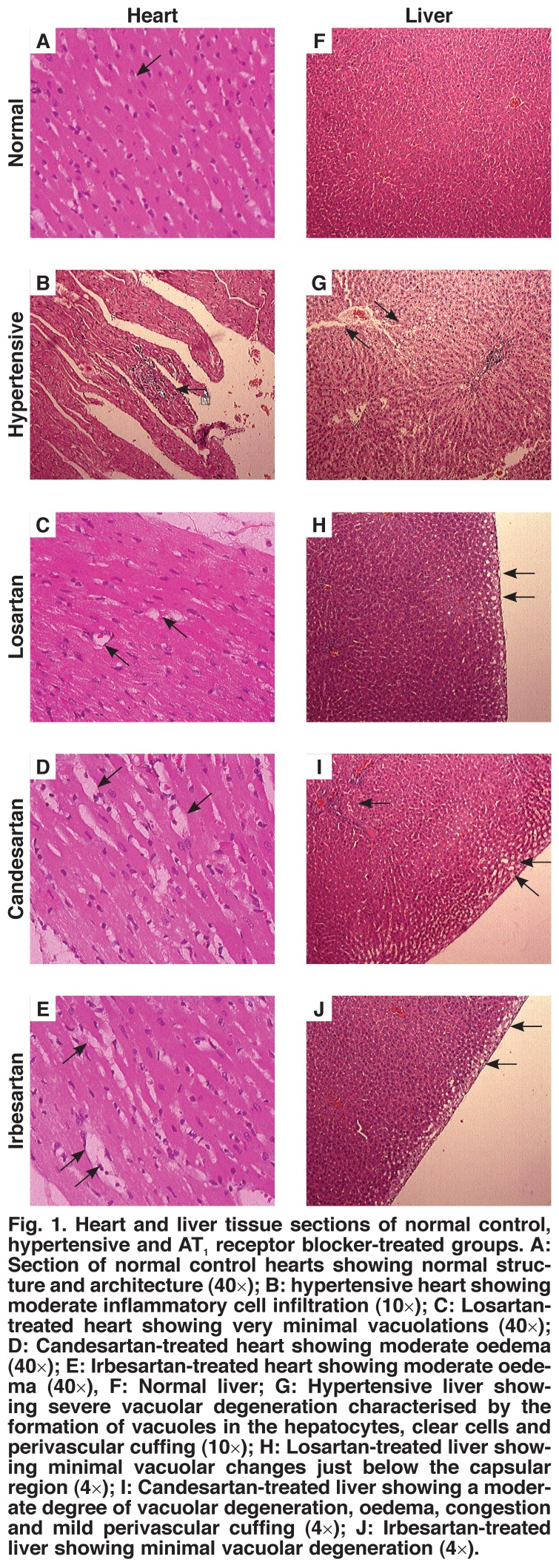
Heart and liver tissue sections of normal control, hypertensive and AT _1_ receptor blocker-treated groups. A: Section of normal control hearts showing normal structure and architecture (40×); B: hypertensive heart showing moderate inflammatory cell infiltration (10×); C: Losartan-treated heart showing very minimal vacuolations (40×); D: Candesartan-treated heart showing moderate oedema (40×); E: Irbesartan-treated heart showing moderate oedema (40×), F: Normal liver; G: Hypertensive liver showing severe vacuolar degeneration characterised by the formation of vacuoles in the hepatocytes, clear cells and perivascular cuffing (10×); H: Losartan-treated liver showing minimal vacuolar changes just below the capsular region (4×); I: Candesartan-treated liver showing a moderate degree of vacuolar degeneration, oedema, congestion and mild perivascular cuffing (4×); J: Irbesartan-treated liver showing minimal vacuolar degeneration (4×).

Histological sections from the normal control, sham-operated rat hearts showed normal structure and architecture. Heart sections of the untreated AABIH rats showed mild to moderate degrees of haemorrhage (accumulation of red blood corpuscles in between the cardiac fibres), mild perivascular fibrosis (fibrous tissue proliferation around the blood vessels), defragmentation of cardiac fibres (loss of striations), congestion (accumulation of red blood cells in the blood vessels in the parenchyma), oedema (separation of cardiac fibres), and mild vacuolations and focal areas of necrosis in one or two areas. The tissue also showed mild lymphocytic infiltration (Fig. 1A, B).

Compared to the untreated AABIH group, the losartan-treated group showed a mild degree of haemorrhage, mild perivascular fibrosis, defragmentation of the cardiac fibres, congestion, oedema and mild vacuolations. The tissue also showed mild lymphocytic infiltration. The candesartan-treated group showed a mild-to-moderate degree of haemorrhage, mild perivascular fibrosis, and defragmentation of cardiac fibres, congestion, oedema, moderate vacuolations and focal areas of necrosis in one or two areas. The irbesartan-treated group showed mild-tomoderate degrees of haemorrhage, mild-to-moderate oedema, and separation of cardiac fibres and congestion (Fig. 1C - E)

The sections of normal control, sham-operated rat livers showed normal structure and architecture. Liver sections of the untreated AABIH rats showed congestion, multifocal areas of necrosis, and dilation of the central vein. There was also a severe degree of degeneration and vacuolations restricted to the border areas below the hepatic capsule, indicating the initial stages of ischaemia (lack of blood supply).

Compared to the untreated AABIH group, the losartan-treated group showed congestion, and dilation of the central vein. There was also a moderate degree of vacuolations in the hepatic parenchyma. The candesartan-treated group showed congestion multifocal areas of necrosis, and dilation of the central vein. There was also a moderate degree of degeneration and vacuolations in the hepatic parenchyma (hydropic degeneration), indicating a moderate degree of ischaemia. The tissue also showed a mild-to-moderate degree of neutrophil and lymphocytic infiltration. The irbesartan-treated group showed congestion, dilation of the central vein, mild haemorrhage and moderate vacuolations in the borders of the hepatic parenchyma, indicating limited ischaemia (Fig. 1F - J).

**Fig. 2.**
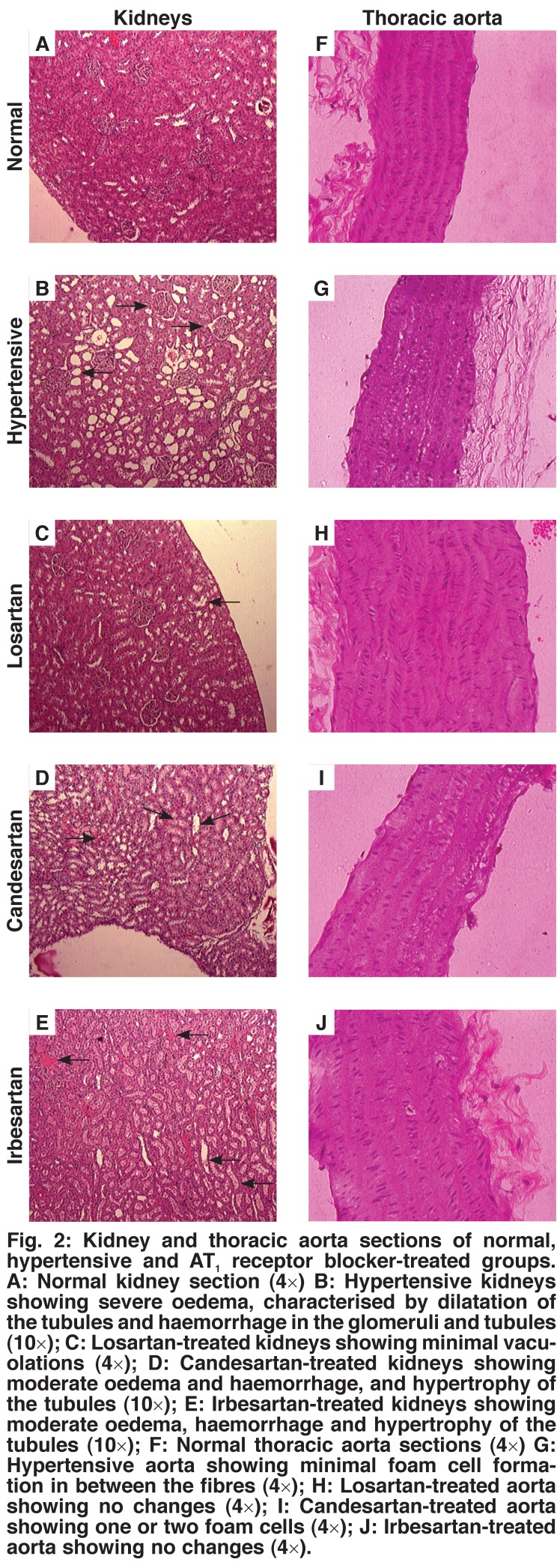
Kidney and thoracic aorta sections of normal, hypertensive and AT _1_ receptor blocker-treated groups. A: Normal kidney section (4×) B: Hypertensive kidneys showing severe oedema, characterised by dilatation of the tubules and haemorrhage in the glomeruli and tubules (10×); C: Losartan-treated kidneys showing minimal vacuolations (4×); D: Candesartan-treated kidneys showing moderate oedema and haemorrhage, and hypertrophy of the tubules (10×); E: Irbesartan-treated kidneys showing moderate oedema, haemorrhage and hypertrophy of the tubules (10×); F: Normal thoracic aorta sections (4×) G: Hypertensive aorta showing minimal foam cell formation in between the fibres (4×); H: Losartan-treated aorta showing no changes (4×); I: Candesartan-treated aorta showing one or two foam cells (4×); J: Irbesartan-treated aorta showing no changes (4×).

The sections of normal control, sham-operated rat kidneys showed normal structure and architecture. The kidney section of the untreated AABIH rats showed oedema, vacuolations in the tubules, moderate to severe haemorrhage and congested vessels. Compared to the untreated AABIH group, the kidney sections of the losartan-treated group showed oedema, vacuolations in the tubules, a moderate degree of haemorrhage, congested blood vessels, and dilatation of vessels and hypertrophy of the tubules. The sections of the candesartan-treated group showed mild oedema, vacuolations in the tubules, a mild-to-moderate degree of haemorrhage, and congested blood vessels. The sections of the irbesartan-treated group showed mild oedema, vacuolations in the tubules, a mild-to-moderate degree of haemorrhage, and congested blood vessels (Fig. 2A - E).

The sections of normal control, sham-operated rat thoracic aorta showed normal structure and architecture. The thoracic aorta section of the untreated AABIH rats showed mild accumulation of foam cells in between the fibres. Compared to the untreated AABIH group, the sections of the losartan-treated group showed no changes. The sections of the candesartan-treated group showed one to two foam cells. The sections of the irbesartan-treated group showed no changes (Fig. 2F–J).

From the results of the histopathological evaluation, it is evident that angiotensin receptor antagonists significantly reduced the histological changes in the target organs such as heart, liver, kidneys and thoracic aorta, compared to the untreated AABIH group. Therefore the AT_1_ receptor blockers have the potential to protect end organs. They were shown in this study to have beneficial effects in the treatment of hypertension, both by decreasing blood pressure and protecting the target organs.

## Discussion

The results of this study demonstrate that blockade of AT_1_ receptors with AT_1_ antagonists reduced the systolic BP significantly, caused an improvement in the myocardial antioxidant reserve (serum catalase and SOD enzyme activity), decreased oxidative stress and reduced the histopathological changes induced in the pressure-overload rat model of AABIH and cardiac hypertrophy.

In our study, abdominal aortic banding was found to have increased systolic BP in a consistent manner, which was sustainable throughout the study period. Constriction of the thoracic or abdominal aorta provides an experimental model of what has been previously described as pressure-overload cardiac hypertrophy. The increased blood pressure proximal to the constriction has been postulated to provide a stimulus for the development of cardiac hypertrophy.^16^

Bardy and co-workers^21^ reported that increased transmural pressure in the aorta might be causing the local generation of Ang-II, which acts synergistically with the transmural pressure to enhance vascular fibronectin expression via the AT_1_ receptor. Furthermore, Bonnet and co-workers^22^ later demonstrated that the AT_2_ receptor mRNA was up-regulated in rat mesenteric arteries after a pressure dose of Ang-II infusion for two weeks, suggesting the involvement of AT1 receptor mediation in this Ang-II effect, because AT1 receptor antagonists inhibited the Ang-II-induced up-regulation of the AT_2_ receptor.

In the aortic banding model, the decreased blood pressure distal to the banding stimulates the kidney to release renin, resulting in increased circulating levels of Ang-II. However, as shown by investigators,^17^,^23^ the fact that the elevation of plasma renin is observed only within a few days of aortic banding does not account for the increased levels of AT_2_ receptor mRNA over three weeks. Because Ang-II binds to the AT_1_ and AT_2_ receptor subtypes with similar affinity,^24^ the contractile response of the aorta to Ang-II seems to be dependent on the relative expression level and/or responsiveness of both receptors. Therefore it seems that the decreased response to Ang-II in the pressure-overloaded aorta is likely to depend on, at least in part, the up-regulation of the AT_2_ receptor.

AT_1_ receptor antagonists dose-dependently attenuated the pressor response to intravenous angiotensin-II^25^-^30^ and reduced blood pressure in animal models of hypertension. They also reduced cardiac hypertrophy and improved haemodynamics in animal models of heart failure.^25^,^31^-^33^ They increased sodium excretion and diuresis, lowered blood pressure and proteinuria, and reduced glomerulosclerosis in rats with chronic renal failure.^34^,^35^

Ang-II receptor antagonists have been thoroughly evaluated for their efficacy in mild, moderate and severe hypertension, and lower BP more effectively than placebo without affecting heart rate.^25^,^27^-^31^ They do so regardless of gender, race or age. Longterm studies have demonstrated that angiotensin II antagonists have comparable efficacy in terms of blood pressure reduction at trough.^28^,^36^-^40^ In the present study, we observed a reduction in systolic BP in the AABIH rats treated with an AT_1_ receptor antagonist, which is in agreement with previously reported studies.

Free radical-scavenging antioxidants such as SOD and catalase are the first line of cellular defense against oxidative injury.41 The observed decrease in levels of these antioxidants in the heart following ischaemia–reperfusion in our study confirms the excessive generation of reactive oxygen species, such as superoxide and hydrogen peroxide, which in turns leads to consumption of these endogenous antioxidants.

It has been well documented that AABIH causes increased oxidative stress in rats, as evidenced by reduction in serum SOD and catalase activities.^42^,^43^ In the present study, we observed that the decreased activities of SOD and catalase in AABIH in the rats were significantly ameliorated by treatment with AT_1_ receptor antagonists. These finding are in accordance with studies reporting that telmisartan had an antioxidant effect in a mouse model of atherosclerosis.^44^ The increase in endogenous antioxidant activities is an indication of structural integrity and protection of the myocardium, which was achieved by treatment with AT_1_ receptor antagonists.

In our studies, we observed significant improvement in endogenous antioxidant activity, as evidenced by the elevation in serum SOD and catalase activity. This is in concurrence with that reported by Khafer and Singal who also showed that treatment with losartan reduced oxidative stress, as indicated by an increase in the redox ratio and decreased lipid hydroperoxide content in the myocardial infarction.^45^ Numerous studies have demonstrated reversal of left ventricular hypertrophy, reduced fibrosis, and improvement in coronary flow and cardiac function following losartan treatment.^46^,^47^

Myocardial antioxidants are dynamic in nature and have been reported to change in various physiological and pathological conditions, including hypertrophy,^48^ exercise^49^ and adriamycin-induced cardiomyopathy.^50^ It is also known that different enzymatic and non-enzymatic antioxidants respond uniquely in a variety of oxidative stress conditions. For example, hypoxia resulted in a reduction in MnSOD and glutathione peroxidase (GPx) activities with no change in catalase activity.^51^ In the pressure overload-induced model of heart failure, only SOD activity was significantly less, with no changes in the GPx and catalase activities.^52^

Studies have reported unique regional differences in non-enzymatic antioxidants in hearts subjected to ischaemia–reperfusion.^53^ The exact stimulus for the altered activity of these enzymes is not known; however, increased free radical formation and/or lipid peroxidation during stress conditions may act as a signal.^54^ Using the rat coronary artery ligation model, studies have reported depressed myocardial endogenous antioxidant reserve and increased oxidative stress associated with poor cardiac function.^55^-^57^

It is important to protect target organs from damage induced by hypertension. This study demonstrates the histological damage caused by hypertension induced by AAB in rats. Mild perivascular fibrosis, oedema and mild lymphocytic infiltration observed in the hypertensive rats concurs with that reported by Chen *et al.*,^58^ along with defragmentation of cardiac fibres, mild-to-moderate degrees of haemorrhage, congestion, mild vacuolations and focal areas of necrosis in one or two areas. All these changes indicate the extent of damage to the heart due to hypertension in this model.

As described in the results, treatment with AT1 receptor antagonists reduced the intensity of cardiac damage, as shown by the mild degree of haemorrhage, mild perivascular fibrosis, defragmentation of cardiac fibres, congestion, oedema and mild vacuolations. Studies by Kumiko and co-workers demonstrated that early and transient treatment with AT_1_ receptor antagonists were effective in the prevention of hypertension-induced end-organ damage.^59^

Sections of liver in the untreated AABIH group showed congestion, multifocal areas of necrosis, and dilation of the central vein. There was also a moderate degree of degeneration and vacuolations restricted to the border areas below the hepatic capsule, indicating early stages of ischaemia. Histological changes following inhibition of the AT_1_ receptors were mild haemorrhage and moderate vacuolations in the borders of the hepatic parenchyma, indicating a decrease in the extent of liver damage or limited ischaemia.

Suppression of D-galactosamine-induced liver injury by the AT_1_ receptor blocker losartan, reported by Chan and co-workers, suggests the protective effect of the AT_1_ receptor blocker.^60^ This is in line with our results, as there were mild-to-moderate degrees of vacuolation and degeneration in the hepatic parenchyma, indicating a moderate degree of ischaemia. Therefore treatment with AT_1_ receptor blockers has therapeutic potential in preventing the histopathological changes observed in target organs of the hypertensive group.

Sections of the kidneys in the untreated AABIH group showed oedema, vacuolations in the tubules, moderate to severe haemorrhage and congested vessels, all of which are signs of renal damage, which is in agreement with an earlier study.^61^ As described in the results, treatment with AT1 receptor antagonists reduced the intensity of damage to the renal tissue, indicated by mild vacuolations in the tubules, a moderate degree of haemorrhage, and congested blood vessels.

Endothelial dysfunction is one of the most important mechanisms involved in the development of atherosclerosis and is present in patients with various cardiovascular risk factors, including hypertension, hypercholesterolaemia and type 2 diabetes, as well as in patients with coronary artery disease. Endothelial dysfunction has important prognostic implications in these groups of patients.^62^,^63^ Blocking the RAS with AT_1_ receptor antagonists clearly ameliorates endothelial dysfunction, an effect that is not totally dependent on BP reduction.

In an elegant study,^64^ resistance arteries obtained from subcutaneous gluteus muscle biopsies from a small group of hypertensive patients and normotensive controls were studied by measuring the endothelium-dependent and independent responses and the cross-sectional area.^64^ Histological sections of the thoracic aorta in the untreated AABIH group showed mild accumulation of foam cells in between the fibres. Treatment with AT_1_ receptor antagonists resulted in protection from this, which may be attributed to the protective effect on vascular endothelium seen in hypertension-induced damage to the vasculature.

## Conclusion

Our study demonstrates that inhibition of the AT1 receptor with AT_1_ antagonists caused an improvement in the myocardial antioxidant reserve and decreased oxidative stress, and prevented pathophysiological alterations associated with hypertension in rats, which was evident in the protection of histological changes observed in the treatment groups. The study also emphasises that modulation of the RAS by AT_1_ receptor blockade is beneficial in preventing target-organ damage in hypertension.
